# Green and facile synthesis of few-layer graphene via liquid exfoliation process for Lithium-ion batteries

**DOI:** 10.1038/s41598-018-27922-z

**Published:** 2018-06-27

**Authors:** Pin-Chun Lin, Jhao-Yi Wu, Wei-Ren Liu

**Affiliations:** Department of Chemical Engineering, Chung Yuan Christian University, Chungli, 32023 Taiwan

## Abstract

A green and facile method using jet cavitation (JC) was utilized to prepare few layer graphene (FLG) derived from artificial graphite delamination without adding any strong acids and oxidants. The JC method not only provides high quality FLG with high yield but also demonstrate excellent electrochemical performance as anode materials for Li-ion batteries. Raman spectroscopy, scanning electron microscopy (SEM), and transmission electron microscopy (TEM) as well as BET isotherms and XPS are carried out in this study. The results of atomic force microscopy (AFM) further revealed that up to 85% of the prepared FLG were less than 10 layers. This exfoliation process happened mainly due to the cavitation-induced intensive tensile stress acting on the layered materials. Electrochemical measurements demonstrate that graphite anode delivered only 240 mAh/g while FLG anode achieved more than 322 mAh/g at 5C rate test. These results indicate that JC method not only paves the way for cheaper and safer production of graphene but also holds great potential applications in energy-related technology.

## Introduction

Owing to excellent properties of graphene reported so far, such as Young’s modulus (>1060 GPa), electron conductivity (6000 S/cm), thermal conductivity (~3000 W/m K), light and inexpensive^1–4^, it is widely used in many applications, like sensors^5^, biomedicines^6^, mechanic resonators^7^, ultra-capacitors^7^, etc. By down-sizing graphite from micro to nano-scale, namely graphene, the applications would future widen to polymer composites, anode materials for Li-ion batteries, supercapacitors, hydrogen storage materials, adsorbers and catalysts^8^.

The synthesis of graphene has been carried out by various methods such as exfoliation and cleavage, ultrasonic exfoliation^9,10^, growth on SiC^11,12^, chemical vapor deposition^13,14^, molecular beam epitaxy, chemical synthesis^15–18^, chemical routes and other methods^19–21^. Mass production of graphene or few layer graphene is being hindered by the expensive cost and environmental threat of its conventional synthesis. For example, disadvantages of chemical vapor deposition are high cost and limitation of area. Chemical routes, so called Hummers’ method, suffered from usage of strong acid and oxidants during oxidation processes. A vast amount of waste acids resulted in environmental pollution and problems. Based on these issues, many researchers focus on green process to synthesize graphene or FLG, such as electrochemical exfoliation^22–24^, ultrasonicaion process^25,26^ and so on. However, many problems, such as low yields and poor quality of as-synthesized FLG still need to overcome.

Recently, there were reported in many kinds of literature by using the high pressure homogenizer^27^ and sonication^28^ to delaminate the graphene or using jet cavitation method and obtained few layer graphene^29^. Yi *et al*. showed the feasibility of delamination by JC for unmodified graphite and other layered materials and investigated the influence of feed concentration and processing time on yield and morphology of the product^30–32^. Moreover, Yi *et al*. demonstrated 10 L batch graphene production by a jet cavitation (JC) method and obtained few-layer graphene with low defect concentration^29^. The processing time, however, was quite long (8 h). Nacken *et al*. reported an environmentally friendly method for graphene production in large quantities (5 L batches) in processing times <3 h by top-down processing of isostatic and unmodified graphite in an industrial high pressure homogenizer^27^. According to the above researches, these solvents are organic-based solvents, like NMP, DMF or acetone in the preparation process, even the surfactants including sodium carboxymethyl cellulose (CMC), non-ionic surfactant TWEEN®80 (TW80) or sodium dodecyl sulfate (SDS) are used. In addition, the production of few-layer graphene was got after using centrifuge. In this paper, we propose a green process by using jet cavitation. Here we use an industrial low temperature ultra-high pressure continuous flow cell disrupter (LTHPD) as a delamination device for delamination of graphite suspension continuously by JC method. In this device the graphite suspension is pumped through a nozzle with a defined flow rate to adjust the system pressure. The cavitation and pressure yields a lateral force that induces exfoliation. Through the combination of velocity gradient-induced shear stress, turbulence-induced Reynolds shear stress, and shear effects that emerged from turbulence and flow-induced collisions, a lateral force is generated causing exfoliation. This normal force caused the bulk material to self-exfoliate to single or few layers through their lateral self-lubricating ability^32^. This method is simple, scalable and does not require toxic chemicals, graphite oxidation or ultrasound post processing to achieve exfoliation. In this study, we propose a green process by using jet cavitation. Here we use an industrial low temperature ultra-high pressure continuous flow cell disrupter (LTHPD) as a delamination device for delamination of graphite suspension continuously. This method is simple, scalable and does not require toxic chemicals, graphite oxidation or ultrasound post processing to achieve exfoliation.

Lithium-ion batteries are among the most widely used energy storage device today. Two-dimensional graphene has drawn much attention because of its admirable properties including conductivity, mechanical strength and high charge carrier mobility which make graphene a suitable electrode material for LIBs^33–36^. As for applications of graphene anode for Li-ion batteries, Lian *et al*.^37^, Jusef *et al*.^38^ and Wang *et al*.^39^ reported graphene nanosheets synthesized by chemical synthesis demonstrate good electrochemical performances as anodes in lithium-ion cells. However, it suffers from problems of poor electronic conductivity, larger hysteresis and irreversible capacity loss in the first cycle due to structural defects and functional groups in graphene nanosheets resulted from oxidation processes. In addition, Haiyan *et al*.^40^ reported the fabrication of anodes for lithium-ion batteries (LIBs) based on graphene nanoflakes. Moreover, the device cycled at 0.5 A/g of current densities present only 200 mAh/g. In 2011, Zhou *et al*.^41^ prepared foam-like graphene as an anode for Li-ion batteries. Its capacity at a current density of 0.2 A/g was about only 200 mAh/g.

To the best of our knowledge, there are few researches concerning electrochemical behavior and measurements of FLG synthesized from jet cavitation process for Li ion battery. Thus, in this study, we will discuss detail characterizations of FLG in terms of SEM, TEM, BET, AFM as well as Raman and XPS to understand surface morphologies, lateral size, thickness distribution, structural defects, functional groups of as-synthesized FLG. The corresponding electrochemical coin tests, such as C/D tests, cycle life as well as differential capacity plots and AC impedances are carried out.

## Results and Discussion

**Figure 1:**
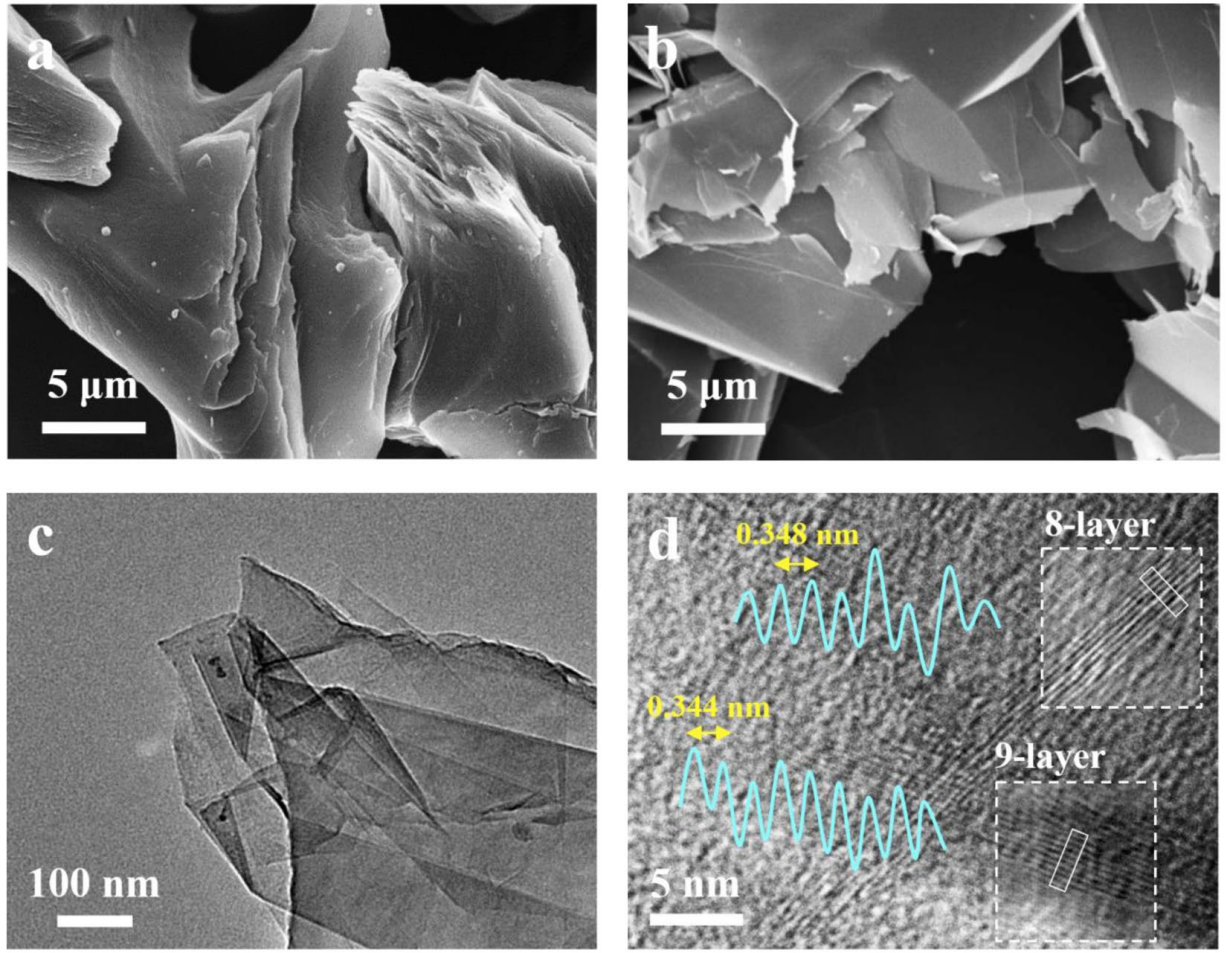
SEM images of (**a**) graphite and (**b**) FLG; TEM image of (**c**) FLG and HRTEM image of (**d**) FLG, in which the lattice planes correspond to (002) planes with an interlayer distance of 0.344–0.348 nm.

Figure 1a shows images of pristine artificial graphite. The lateral size of graphite was about 15 μm. Figure 1b depicts the surface morphology of sample processed in DI water under 200 MPa for 3 cycles. Clearly, the morphology of graphite is thicker and irregular. After liquid exfoliaiton processes, the laterial size of graphite was decreased and delaminated to be few layer graphene (FLG) with thickness <10 nm. This indicates that the initial graphite can be effectively exfoliated into thinner sheets by JCD. Figure 1c shows a typical TEM image of FLG is transparent and folded which coincides well with the typical feature of the reported FLG. Figure 1d shows a typical high resolution TEM (HRTEM) image of FLG which illustrates 8~9 layers of FLG. Figure 1d shows a typical high resolution TEM (HRTEM) image of FLG, in which illustrates 8–9 layers of FLG. The result of HRTEM is corresponded with the AFM. It exhibits HRTEM image of the cross section view of stacked graphene layers. The interplanar distance was measured to be 0.344~0.348 nm corresponding to the spacing of the (002) planes, which is similar to that of graphite (d002 = 0.34 nm)^39^. In this study, FLG is prepared by JCD without chemical intercalation. Thus, d spacing in 002 plane was closed to 0.34 nm.

**Figure 2:**
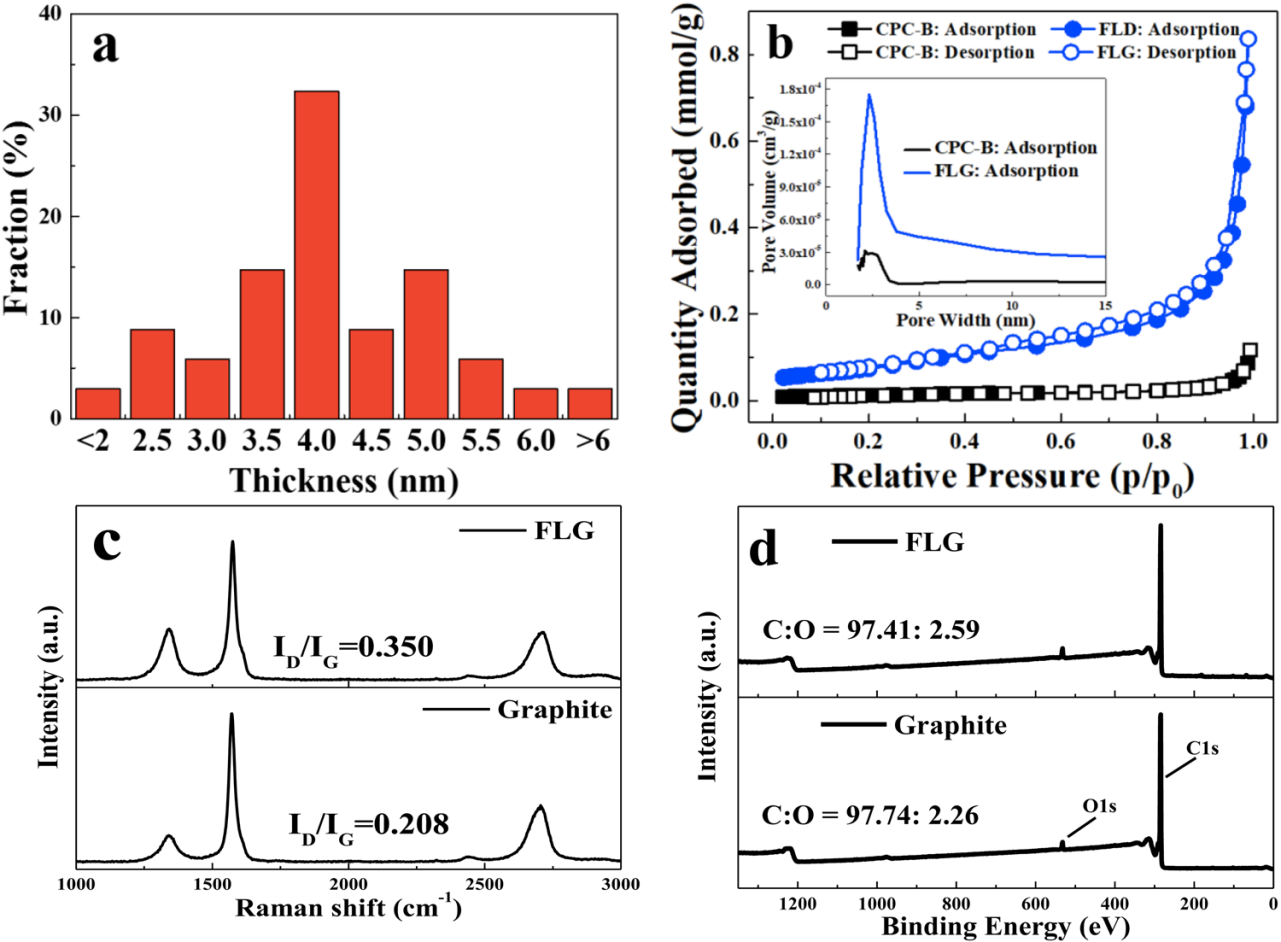
(**a**) Distribution of the thickness of FLG calculated from the obtained AFM analysis; (**b**) Nitrogen adsorption/desorption isotherms of graphite and FLG, inset shows the porosity distribution by Original Density Functional Theory Model; (**c**) Raman spectrum of graphite and FLG; (**d**) XPS spectrum of graphite and FLG.

Figure 2a shows distribution of the thickness of FLG calculated from the obtained AFM analysis under 200 MPa pressure for 3 cycles. For the samples, more than 30 flakes were captured (Figure S1, S2 and S3 and Table S1). They are believed to be monolayers according to the fact that FLG are often measured to be 0.4–1 nm by AFM due to some external factors such as the AFM equipment and substrates^42,43^. The average thickness of FLG was 4 nm, which approached 8 layers of graphene (0.5 nm for one layer of graphene) in Fig. 2a. Up to 85% of the prepared FLG were less than 5 nm thick and most of FLG are belong to 10 layers. The N_2_ adsorption–desorption isotherms of graphene sheets are shown in Fig. 2b. The BET specific surface area of graphite and FLG were measured to be 0.98 and 5.91 m^2^/g, respectively. FLG demonstrated much larger specific surface area, which indicates that the evidence of graphite exfoliation after our processes.

Raman spectra were analysis so as to examine the defect content of graphite and FLG. Typical spectra for graphite and FLG are shown in Fig. 2c. The features in the Raman spectra of graphitic materials are the alleged G band, D band and 2D band appearing at ~1582 cm^−1^, ~1350 cm^−1^ and ~2700 cm^−1^ ^29,44^. The D band is consider the demonstration of the presence of defect and the degree can be estimated by the intensity ratio of the *D* peak to *G* peak (*I*_*D*_/*I*_*G*_). The graphite and FLG of intensity ratio of the *D* peak to *G* peak (*I*_*D*_/*I*_*G*_) increased from 0.208 to 0.350 through the evolution of *I*_*D*_/*I*_*G*_ clearly revealed by comparison of curves. This result demonstrated that defect increased in FLG after exfoliation processes. Furthermore, the oxygen content of graphite and FLG were analysis by XPS is shown in Fig. 2d. Distinct C1s and O1s peaks can be seen from the survey scan. It was demonstrated that the oxygen content of graphite and FLG were 2.26 and 2.59%, respectively. The results indicated that it wasn’t leading to oxidation through exfoliation processes.

**Figure 3:**
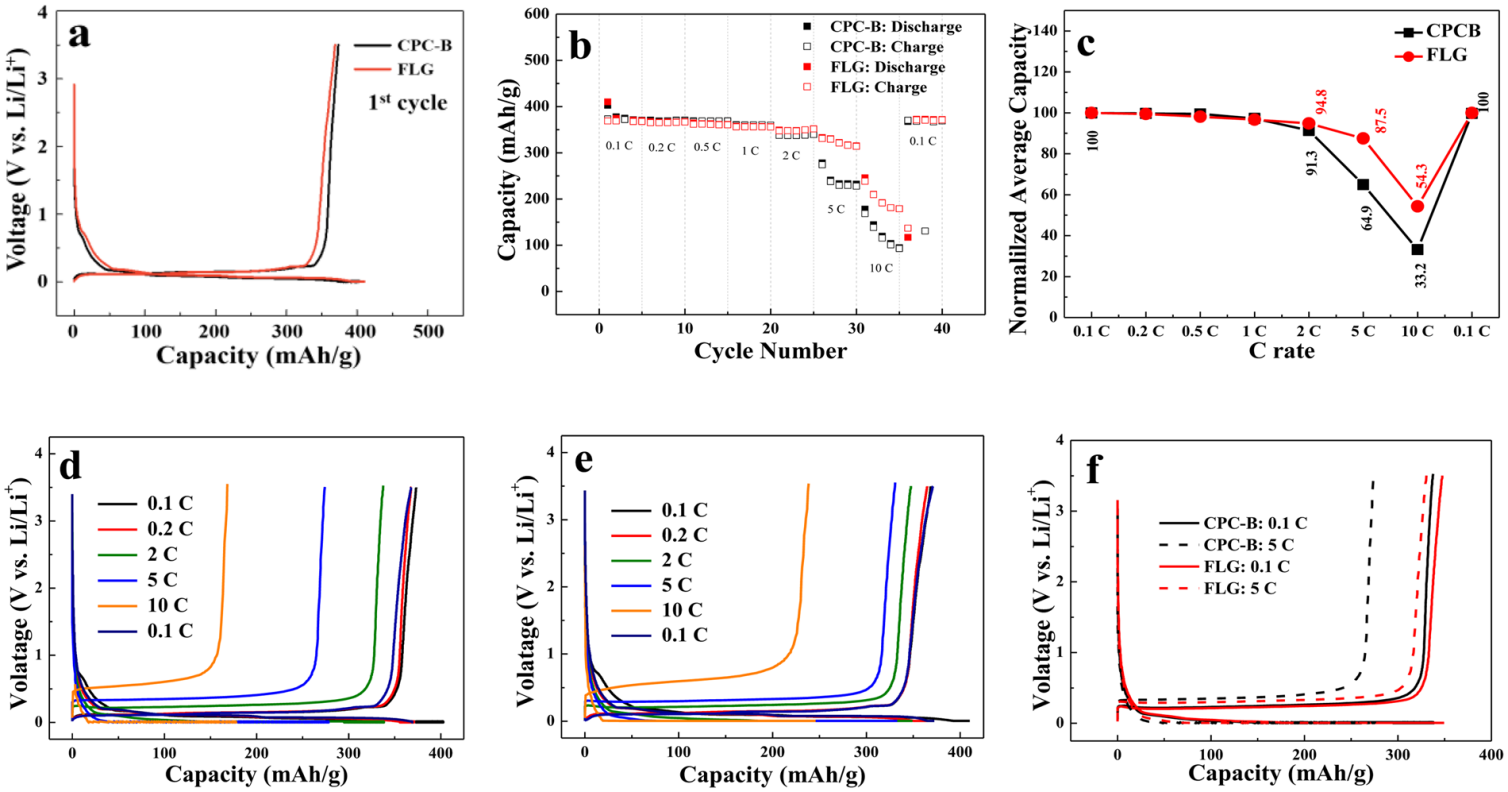
(**a**) The 1^st^ charge/discharge profiles for graphite and FLG at rate 0.1 C; (**b**) Rate performance of graphite and FLG at various current rate from of 0.1 C to 10 C. (**c**) Comparison of graphite and FLG rate performance; (**d**) Representative charge and discharge curves of graphite at various current densities; (**e**) Representative charge and discharge curves of FLG at various current densities; (**f**) Representative charge and discharge curves of graphite at various current densities.

The delaminated of FLG might be applied as potential anode materials in LIBs. In this section, we test the graphite-based (graphite) and graphene-based (FLG) anode materials for lithium ion battery. During lithiation and de-lithiation, the anode may change its shape^45^. However, graphene is known to have good flexibiltiy compared to graphite. It is believed that graphene will retain the integrity of its shape during rapid lithiation process thus, providing better electrochemical performance. Figure 3a shows the galvanostatic discharge profiles (charing/discharge rate = 0.1 C) of graphite and FLG, respectively. The first discharge cycle obtains a reversible capacity of 373 mAh∙g^−1^ and 369 mAh∙g^−1^ for graphite and FLG at a current density of 0.1 C (0.035 A/g), corresponding to a coulombic efficiency of 93% and 90, respectively. The reversibility of FLG is comparable to graphite. The solid electrolyte interface (SEI) layer plays an essential role in the reversibility of the capacity. SEI layer is formed by the reaction between graphite and ethylene carbonate (EC) in the electrolyte. It provides kinetic stability to the cell and permits the use of graphite as anode material at the expense of some inevitable irreversible capacity^46^. Vital properties of energy storage devices such as irreversible capacity loss, cycle life, electrode corrosion, self-discharge rate and safety are highly influenced by the thickness of the SEI layer^47^. Similar to other literature, as-prepared FLG displayed an irreversible capacity ~0.75 V in the initial cycle attributable to the formation of SEI layer and the reaction of Li^+^ ions with residual H or O groups in the carbonaceous materials^37^. Lithium atoms are known to bind quasi-reversibly on the hydrogen terminated edges of graphene. In this study, our few layer graphene is not synthesized by Hummer’s method, namely, reduced graphene oxides. Thus, there is littele functional groups and structural defects in our FLG (As shown in Fig. 2c and d). We believe SEI formation is not a dominate issue for our FLG as anode materials for Li ion batteries^48–50^.

The capacity of FLG was equivalent to the capacity of graphite. The high rate discharge/charge properties are likely to originate in the shortened diffusion distance of lithium ions into the host position of the graphene sheets with fewer layers and porous structure^51^. Rate capability operated at different current rates from 0.1 C to 10 C run for five cycles each (Fig. 3b). As the figure depicts, FLG had average discharge capacities, followed by of 368, 366, 361, 356, 349, 322 and 200 mAh/g at current densities of 0.1, 0.2, 0.5, 1, 2, 5 and 10 C, respectively. An improvement of rate capability was observed with FLG as anodes. The reversible capacity was 368 mAh/g at a 0.1 C rate and maintained at 200 mAh/g even at 10 C, and the Coulombic efficiency of FLG reached to 99%. Compared to the rate capability of graphite and FLG, the specific capacity of FLG was higher than that of graphite at high current densities (2 C and 5 C) shown in Fig. 3c. When the current density goes back to 0.1 C, the specific capacity of the FLG also returns to 371 mAh/g. The rate capability and cycling stability of the graphite and FLG were evaluated by gradually increasing the current rate step-wise from 0.1 C to 10 C, and then returning back to 0.1 C. Figure 3d and e are representative charge and discharge curves of graphite and FLG at various current densities, respectively. These results demonstrated that the prepared graphene sheets with fewer layers have an intensive potential as a candidate of anode materials with high reversible capacity and high rate discharge/charge capability. Increased polarization, as shown in Fig. S4, was observed with increasing current density, particularly with respect to the plateaus at 0.5 V during discharge and 1.0 V during charge that are related to the reversible reaction (Fig. 3f).

**Figure 4:**
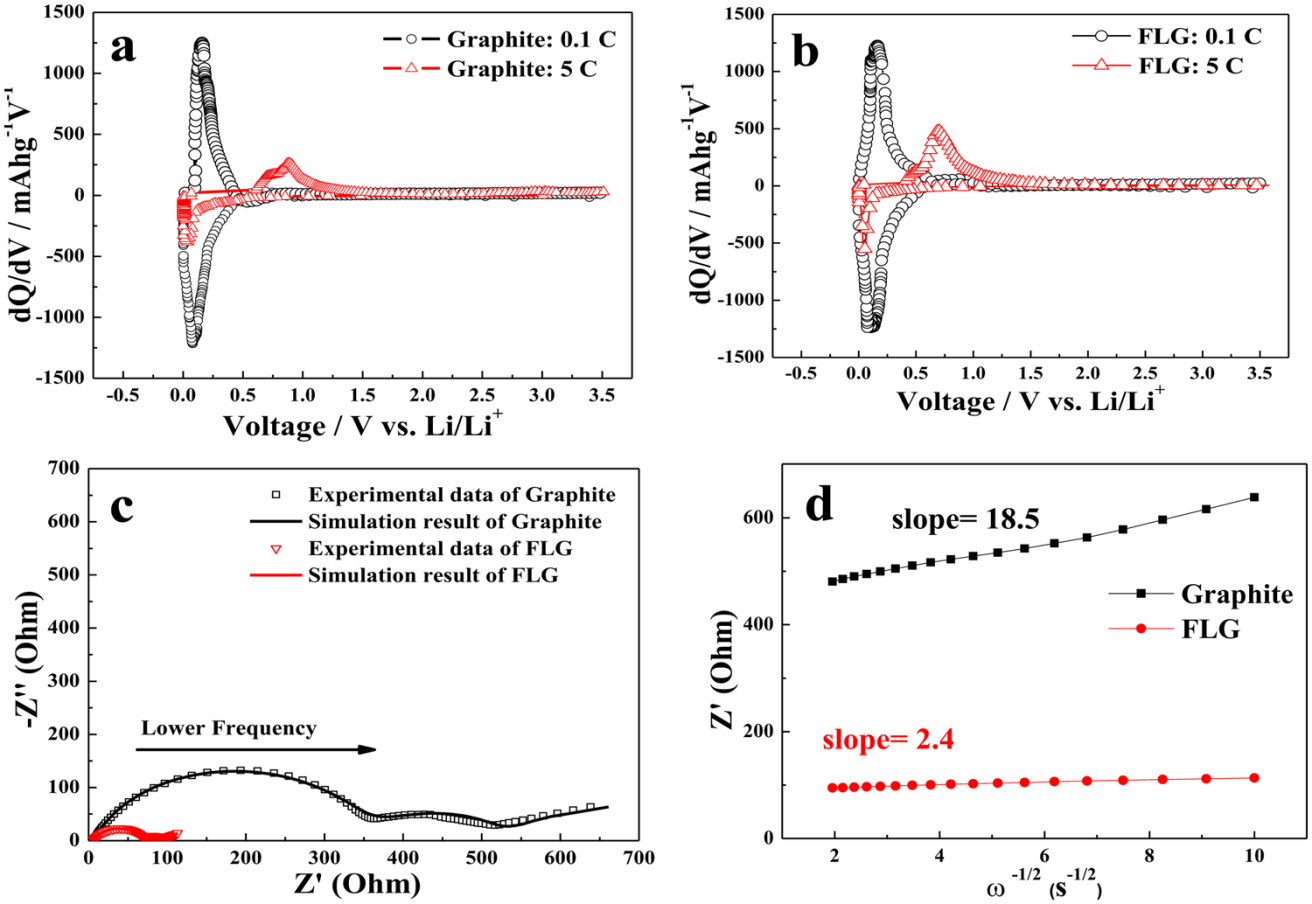
(**a**) AC impedance (inserting the fitted equivalent circuit) of graphite and FLG at the third cycle; (**b**) The relationship lines between Z′ vs. ω^−1/2^ in the low frequency region of graphite and FLG; (**c**) Charge-discharge differential capacity profiles of graphite at 0–3.5 V. (○) 0.1 C and (Δ) 5 C; (**d**) Charge-discharge differential capacity profiles of FLG at 0–3.5 V. (○) 0.1 C and (Δ) 5 C.

Differential capacity plots (dQ/dV) of both graphite and FLG are shown in Fig. 4a and b. These results indicated that the graphite shows comparatively high polarization and the Li^+^ intercalation of FLG was faster than that of graphite. AC impedance of the graphite and FLG electrodes before cycling and after 2.5 cycles (3.5 V vs. Li^+^/Li) are shown in Fig. 4c. As can be seen, two semicircles at the high to medium frequencies and a straight sloping line at low frequency can be observed before cycling in both cases. The first semicircle represents surface films resistance and the second semicircle represents charge-transfer resistance, whereas the straight sloping line is associated with diffusion resistance through the bulk of the active material^52^. An equivalent circuit (inset in Fig. 4c) was used to analyze the measured impedance data, where R_s_ represents the total resistance of electrolyte, electrode, and separator. R_SEI_ and CPE_1_ are the resistance and capacitance, respectively, of the solid electrolyte interface (SEI) formed on the electrode. R_CT_ and CPE_2_ represent the charge-transfer resistance and the double layer capacitance, respectively, and W_1_ is the Warburg impedance related to the diffusion of lithium ions into the bulk electrode^45,52^. The fitting values from this equivalent circuit are presented in Table 1. As can be seen, the R_S_, R_SEI_ and R_CT_ of the graphite electrode are 5.9, 269.9 and 299.9 Ω, respectively, much higher than those of the FLG electrode (R_S_, R_SEI_ and R_CT_ of the FLG electrode are 6.1, 69.1 and 63.2 Ω, respectively), which means that both SEI resistance and charge-transfer resistance are significantly reduced in the presence of exfoliation. It is most likely that a more favorable SEI was formed for the FLG electrode than the graphite, which facilitates the lithium ion transfer at the interface between the electrolyte and the electrode. Besides, the thin characteristic was good for the reaction kinetics and diffusion of Li ion^53^. The Li^+^ diffusion coefficient can be calculated by the following equation^54^:

1 $${\rm{D}}=\frac{{{\rm{R}}}^{2}\,{{\rm{T}}}^{2}}{2{{\rm{A}}}^{2}{{\rm{n}}}^{4}{{\rm{C}}}^{2}{{\rm{\sigma }}}_{{\rm{w}}}^{2}}$$

where *R* is the gas constant (8.314 J K^−1^ mol^−1^), *T* is the absolute temperature (293.15 K) at room temperature, A is the surface area of the electrode (~1.54 cm^2^), *n* is the number of electrons per molecule during oxidization (*n* = 1), *F* is Faraday’s constant (96500 C mol^−1^), and *C* is the concentration of lithium ions (0.001 mol cm^−3^). According to the equation (1), diffusion coefficients of lithium among graphite and FLG were calculated to be 4.39 × 10^−11^ cm^2^/s and 2.67 × 10^−9^ cm^2^/s, respectively. Obviously, diffusivity of Li^+^ in FLG was much higher than graphite. The morphology of electrode materials is addressed as a key factor controlling rapid lithium storage in anisotropic systems such as graphite. The thickness of FLG was found to be 4–5 nm; such morphology favors short diffusion lengths for Li^+^ ions, while the thinner FLG provides connectivity for facile electron diffusion, resulting in high rate performances (Fig. 3b)^53^. Thus, the advantages of thinner FLG for high rate storage performances of battery electrode materials. These results demonstrated that the prepared graphene sheets with fewer layers have an intensive potential as a candidate of anode materials with high reversible capacity, good cycle performance and high rate discharge/charge capability.

**Table 1:** Impedance parameters calculated from equivalent circuit model for graphite and FLG.

Components	Graphite	FLG
R_s_ (Ω)	5.9	6.1
R_SEI_ (Ω)	269.9	69.1
R_CT_ (Ω)	299.9	63.2
W_1_ (Ω)	203.6	17.8
σ_w_	18.5	2.4
D (cm^2^/s)	4.39 * 10^−11^	2.67 * 10^−9^

## Conclusions

In summary, we develope a method to prepare FLG by JCD. SEM and TEM results indicated that it is transparent and folded, which coincides well with the typical feature of the reported FLG. Beyond the HRTEM and AFM presented in this study, the FLG exhibits thickness of the as-synthesized FLG approaches 10 layers of graphene. Furthermore, the obtained graphene were applied as anode material for lithium ion batteries. During the fast charge and discharge rates (5C), graphite delivered ~239.6 mAh/g while FLG exfoliated in DI water achieved ~322.2 mAh/g, respectively. The FLG presented the lower polarization and the Li^+^ intercalation of FLG is faster than that of graphite. The diffusion coefficients of lithium of FLG were increased from 4.39 × 10^−11^ cm^2^/s to 2.67 × 10^−9^ cm^2^/s. It shows great advantages and is thus proved to be a suitable convenient approach for massive production of graphene. As FLG has been successfully produced by this device and these results indicate that it is potential applications of FLG as anode material for lithium ion battery system.

## Methods

### Materials synthesis

**Figure 5:**
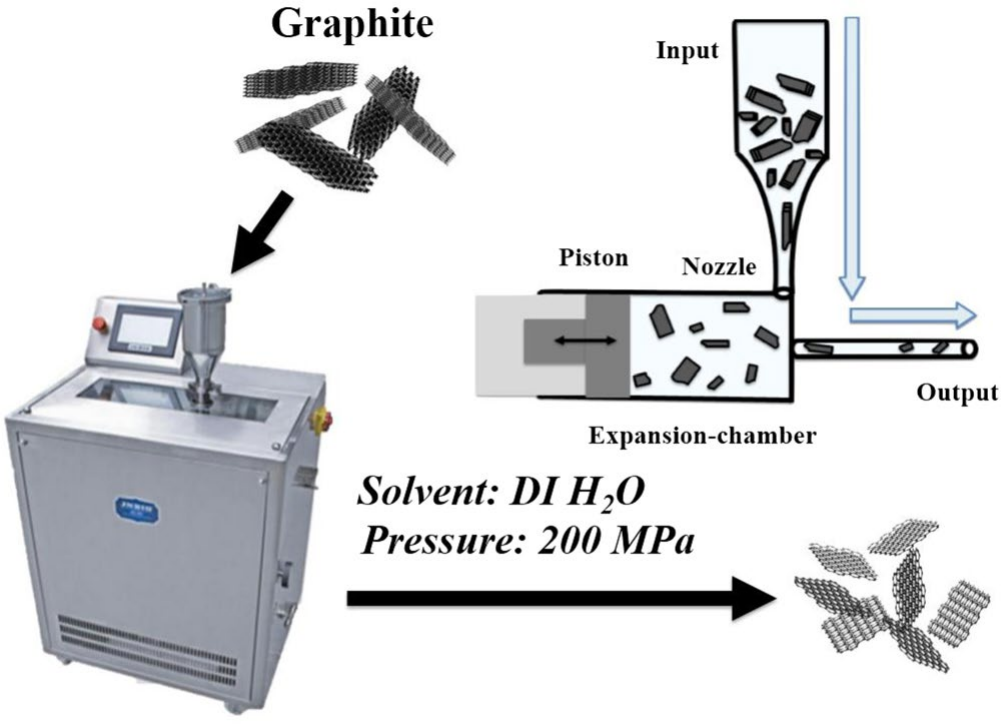
The principle of graphite delamination by JCD. The suspension is pumped through a nozzle and released into an expansion chamber. A counter pressure can be applied to the suspension by moving the piston after the expansion chamber.

The artificial graphite (graphite, purity >99%) was used as feed material for the delamination experiment. All materials were used as supplied without further purification. Deionized water was used for the preparation of all graphite suspensions (10 wt.%). All delamination experiments were carried out in a low temperature ultra-high pressure continuous flow cell disrupter (LTHPD, JNBIO, JN 10C, China). In this device, the graphite suspension is achieved by high pressure forcing the sample through a small orifice at high speed. At the effect of the shearing, hole and impact, graphite of the sample are crushed; substances of the sample are dispersed and emulsified. And the few layer graphene would be kept the suspension at the crash which operated in circulation cooling device at 14–16 °C circulating water bath. The principle and schematic illustration of JC device are shown in Fig. 5. De-ionized water was as a solvent. The graphene production was followed over 3 batch runs and the pressure was in 200 MPa by adapting the piston force through the nozzle. The obtained product of few layer graphene was dried using a vacuum oven at room temperature for battery tests.

### Characterization methods

Raman spectra were obtained with a micro-Raman spectroscopy system; a 532 nm laser was used as the excitation source. Scanning electron microscope (SEM) images were collected by a Hitachi S-4100. Atomic force microscope (AFM) images were captured by a Bruker Dimension Icon. The samples for AFM were prepared by dropping the dispersion directly onto freshly cleaved mica wafer with an injector. Bright-field transmission electron microscope (TEM) and high-resolution TEM (HRTEM) images were taken with a JEM-2100 operating at 200 kV. TEM specimens were made by diluting the suspension in alcohol and pipetting several drops onto holey carbon mesh grid. Brunauer–Emmett–Teller (BET) specific surface area was determined from N_2_ adsorption by using a Micromeritics TriStar 3000 (USA) analyzer at liquid nitrogen temperature. Spectra were all captured at a resolution of 4 cm^−1^, and by averaging 10 scans. X-ray photoelectron spectroscopy analysis was performed with a Thermo Scientific K-Alpha using a monochromated Al Kα X-ray source (1486 eV).

### Electrochemical performance

The electrochemical performance of the products were measured by using CR2032 coin cells. The working electrode was composed of 92 wt.% active materials, 3 wt.% KS-6 (KS-6 is a commercially available synthetic graphite from Timcal® with lateral size of 6 μm (d90)), 1 wt.% Super P (Carbon black, 40 nm) and 4 wt.% PVdF, coating on the 10 μm copper foil, then dried at 120 °C for 8 h in vacuum system to remove the residual water. The electrolyte consisted of 1M LiPF_6_ in ethylene carbonate (EC) and ethyl methyl carbonate (EMC) (1:1 in volume ratio). The discharge/charge test were analysed by AcuTech System in the voltage range of 0.01–3.5 V at room temperature. The Cyclic voltammograms (CV) were measured by CH Instruments Analyser CHI 6273E at a scan rate of 0.001 mVs^−1^ between 0.01 V and 3.5 V, then tested the AC impedance in the frequency range from 1 Hz ~ 100000 Hz in litigation state of 0.001 V.

## Electronic supplementary material

Supporting Information

## References

[1] Soldano C, Mahmood A, Dujardin E (2010). Production, properties and potential of graphene. Carbon.

[2] Lee C, Wei X, Kysar JW, Hone J (2008). Measurement of the elastic properties and intrinsic strength of monolayer graphene. Science.

[3] Frank I, Tanenbaum DM, Van der Zande A, McEuen PL (2007). Mechanical properties of suspended graphene sheets. Journal of Vacuum Science & Technology B.

[4] Cai D, Yusoh K, Song M (2009). The mechanical properties and morphology of a graphite oxide nanoplatelet/polyurethane composite. Nanotechnology.

[5] Schedin F (2007). Detection of individual gas molecules adsorbed on graphene. Nature materials.

[6] Liu Z, Robinson JT, Sun X, Dai H (2008). PEGylated nanographene oxide for delivery of water-insoluble cancer drugs. Journal of the American Chemical Society.

[7] Robinson JT (2008). Wafer-scale reduced graphene oxide films for nanomechanical devices. Nano letters.

[8] El-Kady MF, Shao Y, Kaner RB (2016). Graphene for batteries, supercapacitors and beyond. Nature Reviews Materials.

[9] Lotya M (2009). Liquid phase production of graphene by exfoliation of graphite in surfactant/water solutions. Journal of the American Chemical Society.

[10] Hernandez Y (2008). High-yield production of graphene by liquid-phase exfoliation of graphite. Nature nanotechnology.

[11] Virojanadara C (2008). Homogeneous large-area graphene layer growth on 6 H-SiC (0001). Physical Review B.

[12] Sprinkle M (2010). Scalable templated growth of graphene nanoribbons on SiC. Nature nanotechnology.

[13] Reina A (2008). Large area, few-layer graphene films on arbitrary substrates by chemical vapor deposition. Nano letters.

[14] Wei D (2009). Synthesis of N-doped graphene by chemical vapor deposition and its electrical properties. Nano letters.

[15] Chen J, Yao B, Li C, Shi G (2013). An improved Hummers method for eco-friendly synthesis of graphene oxide. Carbon.

[16] Eigler S (2013). Wet chemical synthesis of graphene. Advanced Materials.

[17] Zhang Y, Fugane K, Mori T, Niu L, Ye J (2012). Wet chemical synthesis of nitrogen-doped graphene towards oxygen reduction electrocatalysts without high-temperature pyrolysis. Journal of Materials Chemistry.

[18] Chen L, Hernandez Y, Feng X, Müllen K (2012). From nanographene and graphene nanoribbons to graphene sheets: chemical synthesis. Angewandte Chemie International Edition.

[19] Lin P-C (2017). Nano-sized graphene flakes: insights from experimental synthesis and first principles calculations. Physical Chemistry Chemical Physics.

[20] Kosynkin DV (2009). Longitudinal unzipping of carbon nanotubes to form graphene nanoribbons. Nature.

[21] Kim KS (2009). Large-scale pattern growth of graphene films for stretchable transparent electrodes. Nature.

[22] Su C-Y (2011). High-quality thin graphene films from fast electrochemical exfoliation. Acs Nano.

[23] Munuera JM (2017). Electrochemical Exfoliation of Graphite in Aqueous Sodium Halide Electrolytes toward Low Oxygen Content Graphene for Energy and Environmental Applications. ACS applied materials & interfaces.

[24] Ossonon BD, Bélanger D (2017). Functionalization of graphene sheets by the diazonium chemistry during electrochemical exfoliation of graphite. Carbon.

[25] Mei X, Ouyang J (2011). Ultrasonication-assisted ultrafast reduction of graphene oxide by zinc powder at room temperature. Carbon.

[26] Khan U (2011). Solvent-exfoliated graphene at extremely high concentration. Langmuir.

[27] Nacken T, Damm C, Walter J, Rüger A, Peukert W (2015). Delamination of graphite in a high pressure homogenizer. Rsc Advances.

[28] Skaltsas T, Ke X, Bittencourt C, Tagmatarchis N (2013). Ultrasonication induces oxygenated species and defects onto exfoliated graphene. The Journal of Physical Chemistry C.

[29] Liang S (2015). Effects of Processing Parameters on Massive Production of Graphene by Jet Cavitation. Journal of Nanoscience and Nanotechnology.

[30] Yi M, Li J, Shen Z, Zhang X, Ma S (2011). Morphology and structure of mono-and few-layer graphene produced by jet cavitation. Applied Physics Letters.

[31] Yi M (2013). Hydrodynamics-assisted scalable production of boron nitride nanosheets and their application in improving oxygen-atom erosion resistance of polymeric composites. Nanoscale.

[32] Yi M, Shen Z, Zhu J (2014). A fluid dynamics route for producing graphene and its analogues. Chinese science bulletin.

[33] Cai X, Lai L, Shen Z, Lin J (2017). Graphene and graphene-based composites as Li-ion battery electrode materials and their application in full cells. Journal of Materials Chemistry A.

[34] Xing Z (2016). One-pot hydrothermal synthesis of Nitrogen-doped graphene as high-performance anode materials for lithium ion batteries. Scientific reports.

[35] Chen F, Yang J, Bai T, Long B, Zhou X (2016). Facile synthesis of few-layer graphene from biomass waste and its application in lithium ion batteries. Journal of Electroanalytical Chemistry.

[36] Sun Y (2017). Comparison of reduction products from graphite oxide and graphene oxide for anode applications in lithium-ion batteries and sodium-ion batteries. Nanoscale.

[37] Lian P (2010). Large reversible capacity of high quality graphene sheets as an anode material for lithium-ion batteries. Electrochimica Acta.

[38] Hassoun J (2014). An advanced lithium-ion battery based on a graphene anode and a lithium iron phosphate cathode. Nano letters.

[39] Wang G, Shen X, Yao J, Park J (2009). Graphene nanosheets for enhanced lithium storage in lithium ion batteries. Carbon.

[40] Sun H (2016). Binder-free graphene as an advanced anode for lithium batteries. Journal of Materials Chemistry A.

[41] Zhou X, Liu Z (2011). Graphene foam as an anode for high-rate Li-ion batteries. IOP Conference Series: Materials Science and Engineering.

[42] Novoselov KS (2004). Electric field effect in atomically thin carbon films. Science.

[43] Nemes-Incze P, Osváth Z, Kamarás K, Biró L (2008). Anomalies in thickness measurements of graphene and few layer graphite crystals by tapping mode atomic force microscopy. Carbon.

[44] Malard LM, Pimenta MA, Dresselhaus G, Dresselhaus MS (2009). Raman spectroscopy in graphene. Physics Reports.

[45] Zuo P, Zhao Y-P (2016). Phase field modeling of lithium diffusion, finite deformation, stress evolution and crack propagation in lithium ion battery. Extreme Mechanics Letters.

[46] Goodenough JB, Kim Y (2009). Challenges for rechargeable Li batteries. Chemistry of materials.

[47] Wang X, Shen W, Huang X, Zang J, Zhao Y (2017). Estimating the thickness of diffusive solid electrolyte interface. Science China Physics, Mechanics & Astronomy.

[48] Zhou H, Zhu S, Hibino M, Honma I, Ichihara M (2003). Lithium storage in ordered mesoporous carbon (CMK-3) with high reversible specific energy capacity and good cycling performance. Advanced Materials.

[49] Allen MJ, Tung VC, Kaner RB (2009). Honeycomb carbon: a review of graphene. Chemical reviews.

[50] Zheng T, Xue J, Dahn J (1996). Lithium insertion in hydrogen-containing carbonaceous materials. Chemistry of materials.

[51] Liang M, Zhi L (2009). Graphene-based electrode materials for rechargeable lithium batteries. Journal of Materials Chemistry.

[52] Aurbach D (2000). Review of selected electrode–solution interactions which determine the performance of Li and Li ion batteries. Journal of Power Sources.

[53] Saravanan K (2009). Storage performance of LiFePO_4_ nanoplates. Journal of Materials Chemistry.

[54] Duan W (2014). Na_3_V_2_(PO_4_)_3_@ C core–shell nanocomposites for rechargeable sodium-ion batteries. Journal of Materials Chemistry A.

