# Supplementary material for: Green and facile synthesis of few-layer graphene via liquid exfoliation process for Lithium-ion batteries
Source: Sci Rep. 2018 Jun 27;8:9766. doi: 10.1038/s41598-018-27922-z (PMC6021450; doi:10.1038/s41598-018-27922-z)
Supplement: Supplementary file 1 — Supporting Information [file 41598_2018_27922_MOESM1_ESM.pdf]

Supporting Information

Green and facile synthesis of few-layer graphene via liquid exfoliation process for lithium ion batteries

Pin-Chun Lin, Jhao-Yi Wu and Wei-Ren Liu*

Department of Chemical Engineering, Chung Yuan Christian University, Chungli, 32023, Taiwan, R.O.C.

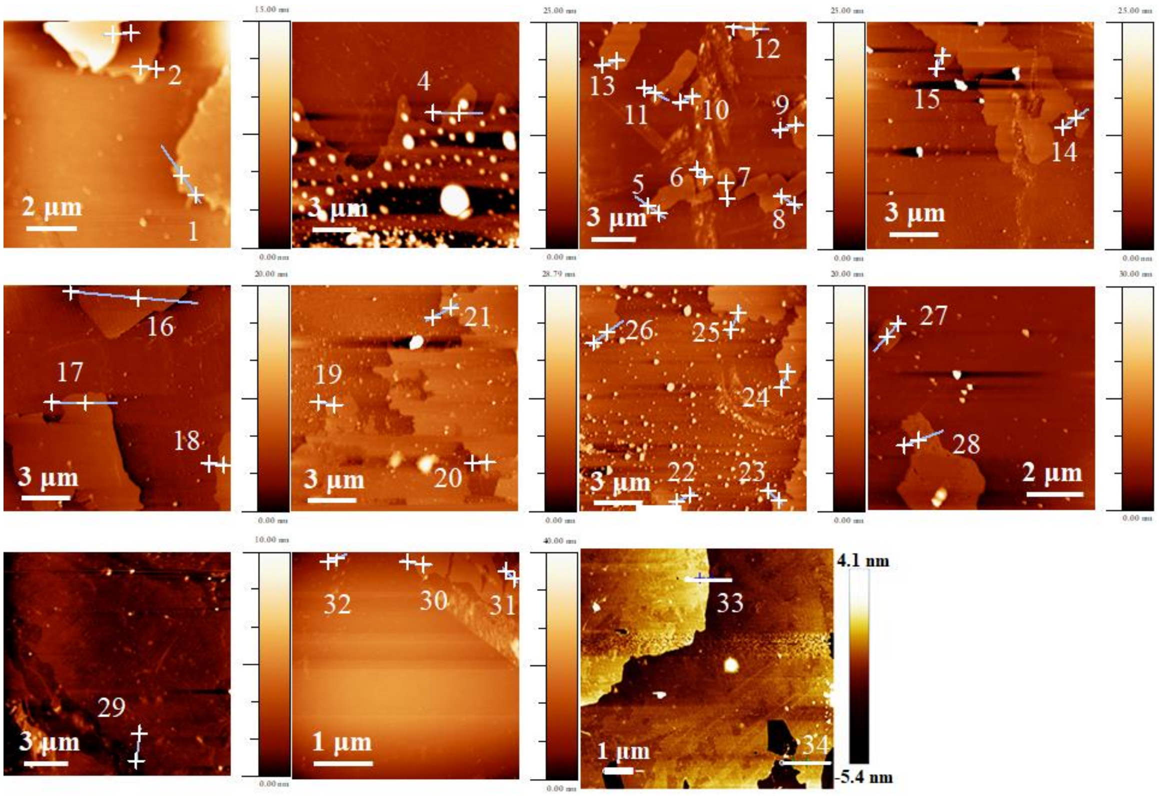

Figure S1 AFM images of FLG.

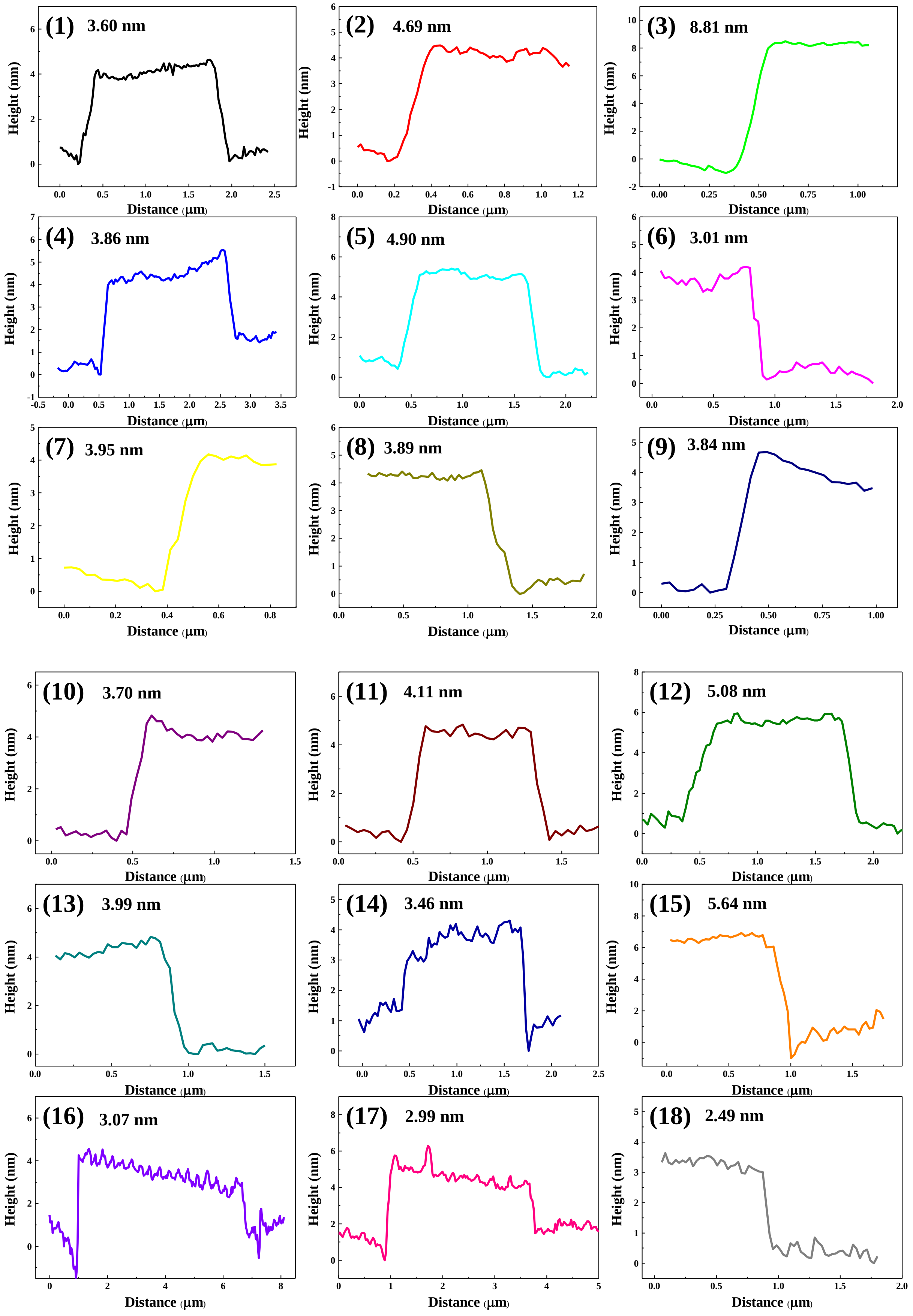
(1) 3.60 nm
(2) 4.69 nm
(3) 8.81 nm
(4) 3.86 nm
(5) 4.90 nm
(6) 3.01 nm
(7) 3.95 nm
(8) 3.89 nm
(9) 3.84 nm
(10) 3.70 nm
(11) 4.11 nm
(12) 5.08 nm
(13) 3.99 nm
(14) 3.46 nm
(15) 5.64 nm
(16) 3.07 nm
(17) 2.99 nm
(18) 2.49 nm
Height (nm)
Distance (μm)

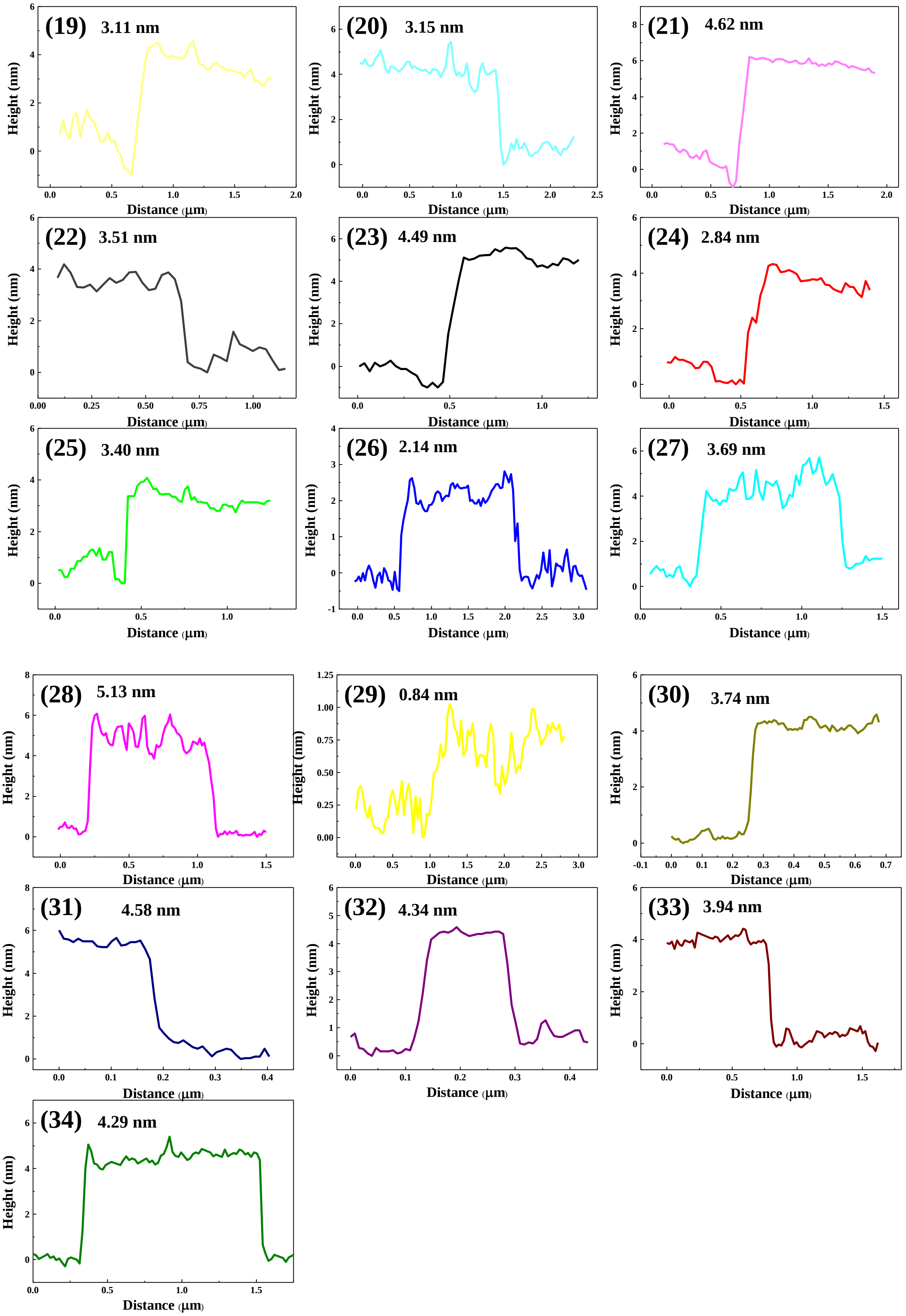

Figure S2 AFM-derived histograms showing the distributions of FLG thickness.

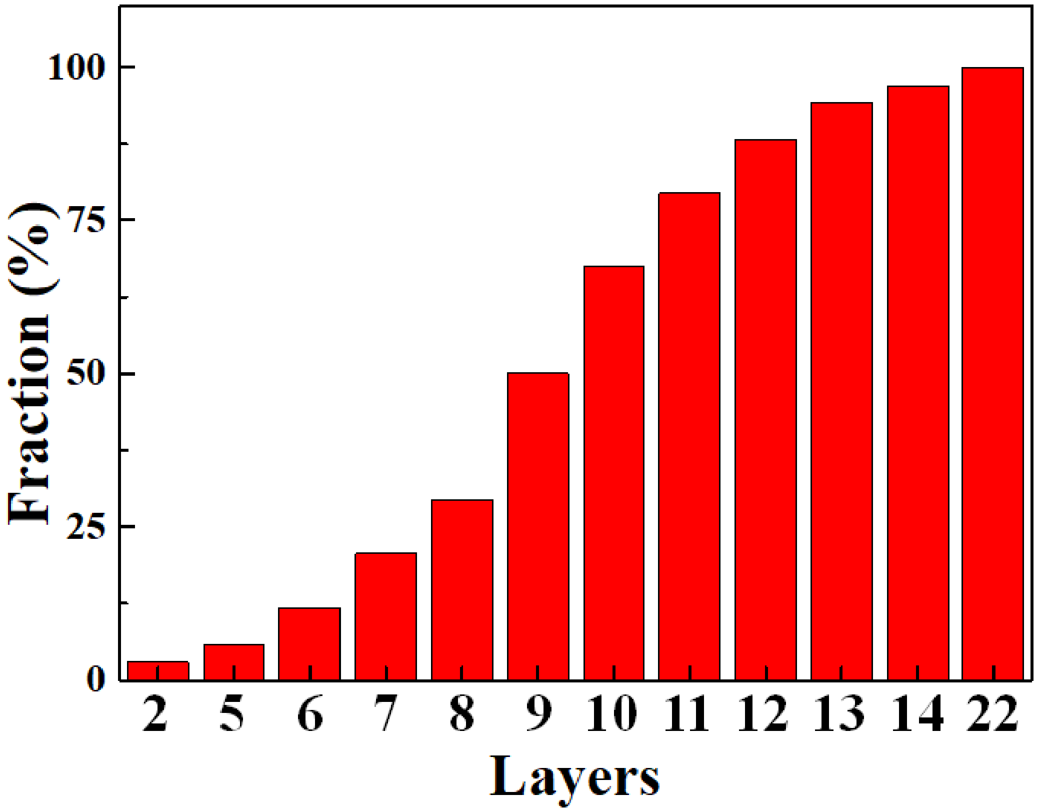

Figure S3 Cumulative distribution of the number of graphene layers per flake calculated from the obtained AFM distribution for FLG.

Table S1 AFM showing the distributions of FLG thickness and FLG layers.

No.	Thickness (nm)	Layers	No.	Thickness (nm)	Layers	No.	Thickness (nm)	Layers
1	3.6	9	13	3.99	10	25	2.4	6
2	4.69	12	14	3.46	9	26	2.14	5
3	8.81	22	15	5.64	14	27	3.69	9
4	3.86	10	16	3.07	8	28	5.13	13
5	4.9	12	17	2.99	7	29	0.84	2
6	3.01	7	18	2.49	6	30	3.74	9
7	3.95	10	19	3.11	8	31	4.58	11
8	3.89	10	20	3.15	8	32	4.34	11
9	3.84	9	21	4.62	11	33	3.94	10
10	3.7	9	22	3.51	9	34	4.92	12
11	4.11	10	23	4.49	11			
12	5.08	13	24	2.84	7			
